# Hematopoietic and Mesenchymal Stem Cells for the Treatment of Chronic Respiratory Diseases: Role of Plasticity and Heterogeneity

**DOI:** 10.1155/2014/859817

**Published:** 2014-01-19

**Authors:** Massimo Conese, Donatella Piro, Annalucia Carbone, Stefano Castellani, Sante Di Gioia

**Affiliations:** ^1^Department of Medical and Surgical Sciences, University of Foggia, Viale L. Pinto 1, 71122 Foggia, Italy; ^2^Medical Genetics Laboratory, Fondazione IRCCS Ca' Granda Ospedale Maggiore Policlinico, Via Commenda 12, 20122 Milan, Italy

## Abstract

Chronic lung diseases, such as cystic fibrosis (CF), asthma, and chronic obstructive pulmonary disease (COPD) are incurable and represent a very high social burden. Stem cell-based treatment may represent a hope for the cure of these diseases. In this paper, we revise the overall knowledge about the plasticity and engraftment of exogenous marrow-derived stem cells into the lung, as well as their usefulness in lung repair and therapy of chronic lung diseases. The lung is easily accessible and the pathophysiology of these diseases is characterized by injury, inflammation, and eventually by remodeling of the airways. Bone marrow-derived stem cells, including hematopoietic stem/progenitor cells (HSPCs) and mesenchymal stromal (stem) cells (MSCs), encompass a wide array of cell subsets with different capacities of engraftment and injured tissue regenerating potential. Proof-of-principle that marrow cells administered locally may engraft and give rise to specialized epithelial cells has been given, but the efficiency of this conversion is too limited to give a therapeutic effect. Besides the identification of plasticity mechanisms, the characterization/isolation of the stem cell subpopulations represents a major challenge to improving the efficacy of transplantation protocols used in regenerative medicine for lung diseases.

## 1. Introduction

Chronic lung diseases, such as asthma and chronic obstructive pulmonary disease (COPD), represent a very high social burden. For example, COPD is the fourth leading cause of death in the world and by the year 2020 it is expected to be the third leading cause of death and the fifth leading cause of disability [1]. The current therapeutic approaches to COPD mainly involve the control of symptoms, without a significant change in the natural history of the disease. Corticosteroids are a mainstay of treatment for asthma and COPD however, some of the asthmatic patients and most of the COPD subjects are steroid resistant [2]. Thus, novel pharmacological and/or innovative therapeutic approaches are being sought for asthma and COPD. Another orphan disease which heavily involves the lung is cystic fibrosis (CF), the most common lethal autosomal-recessive disorder in the Caucasian population. The average life span of CF patients is around 40 years and obviously CF is also the target of novel medications that may alleviate the pulmonary symptoms [3].

In recent years, numerous reports have shown that bone marrow (BM)-derived stem and progenitor cells can give rise to differentiated cells of multiple nonhematopoietic organs including the lung, a phenomenon often referred to as “plasticity” [4]. Based on these initial results, BM-derived stem/progenitor cells are being exploited in the clinic for their therapeutic potential in chronic lung diseases, such as COPD, pulmonary fibrosis, and pulmonary hypertension (reviewed in references [5–9]). However, so far, it is unresolved which subpopulation of BM cells is capable of giving rise to cells of nonhematopoietic lineages. In this paper, we revise the overall knowledge about the engraftment of exogenous marrow-derived stem/progenitor cells into the lung, as well as their usefulness in lung repair and therapy of chronic lung diseases, such as CF, asthma, and COPD. All these diseases are characterized by a chronic inflammatory process which eventually leads to a remodelling process of the airways, making them an attractive target for BM-stem/progenitor cell-based therapy.

## 2. Inflammation and Remodelling of the Airways in Chronic Lung Diseases

Chronic obstructive pulmonary disease (COPD) manifests in two clinical phenotypes: bronchitis and emphysema. Lung tissue in a patient with chronic bronchitis shows thickened bronchial walls with luminal narrowing and mucous plugging or mucopurulent debris within the airways. Microscopically, these gross findings correspond to goblet cell hyperplasia, thickening of the subepithelial basement membrane, bronchial wall fibrosis, and hyperplasia of the subepithelial seromucinous glands. Patients with chronic bronchitis have increased neutrophils and macrophages in the bronchoalveolar lavage fluid (BALF) compared to healthy control subjects [10, 11]. Pulmonary emphysema is characterized by enlargement of airspaces distal to the terminal bronchiole, the destruction of alveolar walls, and loss of the alveolar unit. The main etiological factor in COPD is cigarette smoking which, upon interaction with genetic host factors, determines the pathologic triad of COPD: persistent inflammation, protease-antiprotease imbalance, and oxidative stress. This triad results in mucous/goblet cell metaplasia and hyperplasia, mucous hypersecretion, fibrosis, smooth-muscle alterations, and lung-tissue destruction [12].

Asthma is an allergen-driven chronic inflammatory disorder of respiratory airways induced by cellular mechanisms that produce increased levels of reactive oxygen species (ROS) [13]. In predisposed individuals, elevated ROS production can ensue in allergic inflammation, characterized by IgE-dependent activation of mucosal mast cells and infiltration of eosinophils that is orchestrated by increased numbers of activated CD4^+^ Th2 lymphocytes [14]. Airway wall remodelling in asthma is characterized by structural alterations including epithelial damage, subepithelial reticular basement membrane thickness, subepithelial fibrosis, airway smooth muscle hypertrophy and hyperplasia, and mucous gland hypertrophy [15].

CF is due to mutations in a single gene, the CF transmembrane conductance regulator (CFTR), which is a chloride channel expressed on the apical membrane of epithelial cells [16]. As a consequence, an impaired secretion/absorption of ions and water ensues in a number of different organs. Although CF is a multiorgan disease, the lung pathology is the one mainly responsible for patient morbidity and mortality. In the airways, the imbalanced secretion of chloride combined with hyperabsorption of sodium (due to hyperactivity of the epithelial sodium channel [ENaC]) determines the formation of dehydrated thick mucus which is the trigger for bacterial infection and a subsequent neutrophil-dominated inflammatory response [17, 18]. Neutrophils attracted in the airways are thought to determine more damage than help. Bacteria target neutrophils by their own proteases, causing apoptosis, secondary necrosis, and release of proinflammatory and toxic products [19]. Besides airway epithelial cells and neutrophils, macrophages are also being intensively studied for their contribution to the pathophysiology of CF lung disease [20]. Murine alveolar macrophages have been shown to express CFTR and their defect in the acidification of lysosomes is thought to have a role in the lack of an appropriate host response to opportunistic bacterial pathogens [21, 22]. The chronic inflammatory process eventually leads to a remodelling process of the airways [23, 24], mediated by metalloproteinases secreted by inflammatory cells [25].

Due to the intense production of inflammatory signalling molecules able to recruit circulating cells derived from bone marrow to the site of injury, it is tempting to speculate that BM progenitor/stem cells might be attracted through chemotaxis to the damaged lung. Presently, the role of BM-derived stem and progenitor cells in the repair of an injured lung in the postnatal life has not been completely elucidated and is at least a controversial field, as we shall point out below. Moreover, it remains to be established whether BM-derived cells can contribute to the pool of endogenous lung progenitor cells during regeneration. In mouse models, Krause and coworkers established the presence of marrow-derived lung cells up to 11 months after transplantation [26], and more recent work which applied stringent molecular biology and morphometric assays showed also prolonged engraftment (at least for 4 months) (see, e.g., [27]).

A study on hyperoxia-induced bronchopulmonary dysplasia in a mouse model suggests that BM-derived mesenchymal stromal (stem) cell (MSC) treatment can increase both the overall number of bronchioalveolar stem cells (BASCs) and the number of junctions scoring positive for these progenitors [28]. On the other hand, one recent study using a model of postpneumonectomy with compensatory lung growth in mice found that no donor BM-derived cells engrafted as airway or alveolar epithelial cells at the bronchoalveolar duct junction, a site where putative progenitor cells have been found [29]. The use of different lung injury models may be the reason for these divergent results, suggesting that the contribution of BM-derived cells to the endogenous pool of lung progenitor populations may vary with the lung disease, the type of injury and the repair process.

## 3. Engraftment of Marrow-Derived Stem Cells and Relevance for Lung Repair

In 2001, Krause and colleagues [26] published compelling evidence on the fact that a rare population of single BM-derived cells was able to repopulate the hematopoietic system and generate nonhematopoietic cell types in multiple tissues including epithelial cells of the liver, lung, skin, and gastrointestinal tract. Subsequently, several studies in murine models have demonstrated the ability of marrow-derived hematopoietic stem/progenitor cells (HSPCs) to home to the lung and engraft as airway and respiratory epithelial cells [30–33]. Others have shown that blood-borne stem cells may contribute to lung tissue in human recipients of bone marrow or lung transplantation [34–36]. Besides HSPCs and MSCs, bone marrow contains endothelial progenitor cells (EPCs), and also a marrow-derived circulating cell with fibroblast-like features, termed fibrocyte, has been described. Numerous studies performed in various animal models have demonstrated the ability of MSCs, EPCs, and fibrocytes to home to the lung and differentiate into a variety of cells types, including epithelial, endothelial, fibroblasts, and myofibroblast cells (reviewed in [8, 9, 37]).

From these studies, a caveat emerged and that is that the conversion of marrow-derived stem/progenitor cells into nonhematopoietic cells is very limited in the absence of lung injury, as also shown by further studies in mice [38, 39], indicating that they are not involved in the maintenance of the steady-state homeostasis of lung architecture and function.

Homing to the lung and engraftment of bone marrow-derived stem/progenitor cells into the airways is a very inefficient process. Most of the studies showed that only a very small proportion, that is, <0.01–0.025% of lung epithelial cells, is derived from bone marrow-derived cells (reviewed in [40]). However, using mouse models, besides some authors reporting very low numbers of lung epithelial cells that are marrow-derived [26, 31, 38, 41], there are others who find a higher percentage of BM-derived lung epithelial cells [42, 43], whereas in contrast, some authors were not able to identify any marrow-derived respiratory epithelial cells [44–47]. Explanations to these contrasting results include problems associated with detection of marrow-derived lung epithelial cells, animal models closer to the *in vivo* situation and route of administration, and heterogeneity of administered cells [48]. Detection of rare marrow-derived epithelial cells presents a great challenge and requires extremely sensitive and specific detection techniques [49, 50]. The major common problems to be addressed are (1) overlay of BM-derived cells with epithelial cells, ruled out by CD45 staining and confocal or deconvolution microscopy; (2) lack of identification of marrow-derived epithelial cells by use of lung epithelial specific markers; and (3) lack of adequate positive and negative controls.

From different studies in mice [27, 51, 52], it emerged that transtracheal delivery was more efficacious in enhancing bone marrow cell retention in the lung than the intravenous route. The systemic delivery resulted in null or fewer cells in the lung, with large amounts of these cells in the spleen, liver, and bone marrow [27, 52]. In an effort to draw closer to the pathophysiology of CF lung disease, we have demonstrated that in a murine model mimicking the early phases of bacterial infection and airway remodelling occurring in CF patients, the local administration of lineage negative (lin^−^) Sca-1^+^ HSPCs resulted in a limited (~1%) transformation of HSPCs into respiratory epithelial cells [52]. Thus, the conversion of HSPCs into pulmonary epithelial cells looks like it is independent from the animal model used and it is more likely linked to selected populations which have inherent hurdles in engrafting to the lung and transforming into epithelial cells.

The mechanism by which marrow cells acquire not only the phenotype but also the protein expression of epithelial cells has been investigated and is presently still under discussion [49, 50]. There are multiple potential mechanisms by which BM-derived cells could engraft as respiratory epithelial cells. One possibility is that the bone marrow hosts a previously unsuspected population of epithelial stem/progenitor cells that differentiate into hematopoietic stem cells and into epithelial cell lineages, the so-called multipotent adult progenitor cells (MAPCs), as shown in the mouse [53].

Similar to the previous possibility, a second one is that there are epithelial progenitor cells in the bone marrow that are capable of engraftment as epithelial cells, but not as hematopoietic cells, as particularly shown in mice [54–56].

A third mechanism may be transdifferentiation of committed HSPCs after they are reprogrammed by the microenvironment in the injured lung. So far, no data have been published that directly support this hypothesis.

A fourth option for plasticity could be fusion of a BM-derived cell with a mature pneumocyte or an airway cell lining the bronchiole such that it then undergoes nuclear reprogramming. Although murine marrow-derived lung epithelial cells can appear without any evidence of cell fusion [57, 58], others have demonstrated that they can be derived in part from cell fusion events in mice [59].

A fifth mechanism suggested is the consumption by marrow cells of lung-derived microvesicles. It has been proposed that the injured lung is capable of inducing epigenetic modifications of marrow cells, influencing them to assume phenotypic characteristics of lung cells. Although fusion events have been considered rare in this context, Aliotta and colleagues demonstrated in mice that lung-derived microvesicles are consumed by marrow cells inducing the expression of lung-specific genes in marrow cells, augmenting their capacity of becoming epithelial cells upon transplantation [60]. Both direct transfer of pulmonary epithelial cell-specific mRNA to marrow and induced transcription of pulmonary epithelial cell-specific mRNA in marrow cells were hypothesized by using murine BM cells cocultured with rat lung [61].

**Figure 1:**
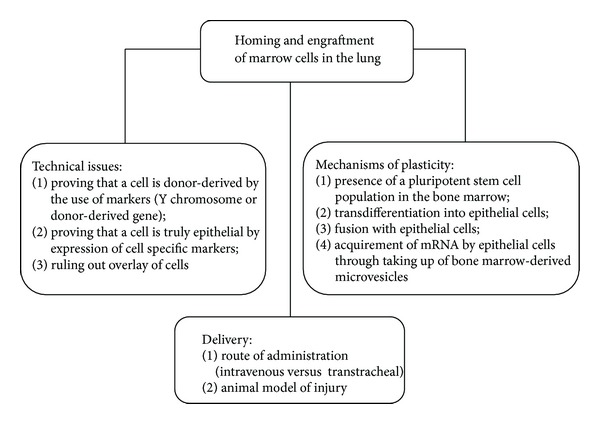
Still debated issues about homing and engraftment of marrow cells in the lung.

Although no definitive data are available for these different possibilities, it is likely that engraftment of marrow-derived epithelial cells occurs via multiple different mechanisms, depending on the entity of damage and which subset of marrow cells is involved. The still unresolved issues about homing and engraftment of marrow cells in the lung are summarized in Figure 1.

## 4. Relevance of Marrow-Derived Stem/Progenitor Cells for Lung Diseases

On the basis of the data currently available, engraftment of airway or alveolar epithelium by stem/progenitor cells originating from the bone marrow is now viewed to be a rarer occurrence than previously described and of unclear physiologic and therapeutic significance [37, 62]. It is now thought that the positive outcome obtained in animal models is due to paracrine factors which may enhance the regeneration of epithelial or endothelial cells, or modulate inflammatory and immune responses [63].

Circulating EPCs can contribute to regeneration of pulmonary vasculature in a mouse model for emphysema in response to lipolysaccharide-induced lung injury [64] and have been investigated in patients with pulmonary arterial hypertension (PAH) in two small clinical trials [65, 66]. The results demonstrated EPC administration to be safe and led to a clinical study (Pulmonary Hypertension: Assessment of Cell Therapy, PHACeT, as registered on ClinicalTrials.gov (NCT00469027)) being initiated to assess the safety of a randomized controlled trial of autologous EPCs and eNOS gene transfer for idiopatic PAH [67].

Increasing evidence obtained, particularly in mouse models, suggests that circulating fibrocytes can contribute to the pathophysiology of fibrotic lung diseases [68–74], pulmonary hypertension [74, 75], and severe asthma [74–76], and thus may be a potential therapeutic target. Further research is needed in order to more fully understand their role in pulmonary fibroses and chronic lung diseases. So far, their use as therapeutic agents in humans with chronic lung diseases has not been conceived yet.

There is an increasing number of studies demonstrating a functional role of MSCs in rodent models of acute lung inflammation and fibrosis in the absence of significant lung engraftment both by transtracheal [77, 78] and systemic administration [79, 80]. MSCs have been found to exert profound suppressive effects on immune cells and pathways [81–84] and have demonstrated both safety and efficacy features in phase 1 and 2 trials in immune-mediated diseases such as graft-versus-host disease (GVHD) [85] and Crohn's disease [86]. In addition, MSCs are being explored for clinical application in renal, cardiovascular diseases, and osteogenesis imperfecta [87]. In the field of chronic lung disease, the administration of MSCs has had therapeutic effects in preclinical animal models of asthma and COPD [63, 88, 89]. On the other hand, initial studies on CF mouse models using poorly characterized BM-derived donor cells demonstrated very low levels of engraftment, likely insufficient to give any positive outcome in the lung disease [90–92]. It is worth noting that studies on asthma and COPD looked at the immunoregulatory role of MSCs, while those on CF were carried out for another purpose, that is, the resumption of the basic ion transport defect at the level of the airway epithelium. Thus, further studies on the role of MSCs in modifying the immunological and inflammatory asset in CF animal models are warranted.

There are scarce data in humans confirming the clinical potential of BM-derived stem cells in chronic lung diseases [93]. The most compelling example come from the use of MSCs in COPD patients [89]. In one unicentric study, the systemic administration of autologous bone marrow mononuclear cells in patients with advanced COPD proved to be well tolerated [94]. Prochymal, human MSCs derived from adult healthy donors, was administered systemically in four doses to patients with moderate-to-severe COPD in a placebo-controlled randomized multicentre trial, demonstrating to be safe [95].

## 5. Heterogeneity of Marrow-Derived Stem/Progenitor Cells

Despite many efforts to isolate a pure hematopoietic progenitor cell population based on patterns of gene expression in combination with differences in cell size, density, and the uptake of fluorescence probes such as rhodamine-123, the resulting cell population remains heterogeneous, as shown in different species (mouse, man, rhesus, and miniature swine) [96–103]. More recent transplantation studies in mice [104–107] recognized the existence of HSPCs with different behaviour in terms of long-, intermediate-, and short-term engraftment patterns. Even in functionally identified stem cell populations, cellular and molecular properties and behaviours vary, as evidenced in mouse models [104, 108]. In particular, Dykstra and colleagues [104] demonstrated that repopulation kinetics, long-term self-renewal potential, and myeloid versus lymphoid bias are intrinsic HSPC properties, stably inherited to their HSPC offspring. More recently, Challen and colleagues [109] have confirmed these findings, demonstrating that the side population (SP) phenotype can separate myeloid from lymphoid phenotype in mice. Whether this heterogeneity is an inherent HSPC property and possibly even necessary for their function or whether it simply reflects our inability to purify them to homogeneity is currently unknown [110]. Answering this question will require improved purification approaches, but many studies do point to intrinsic stem cell heterogeneity both in human and mouse cells [104, 111–113]. Recent methods to study stem cells include long-term single-cell imaging [114] and clonal analysis [115]. Regardless of the underlying mechanism(s), these findings indicate that the HSPC lineage bias is governed more by a stable intrinsic epigenetic program than by extrinsic signals *in vivo*. However, appropriate environmental signals are nevertheless essential for the maintenance of both HSPC self-renewal and the lineage bias program (see below).

Of the other stem cell populations present in the bone marrow, MSCs are also endowed with heterogeneity in humans and mice [116]. The MSC potency (defined as the trilineage potential to exhibit adipo-, chondro-, and osteogenesis) is unevenly distributed in an apparent homogeneous population in human BM-derived cells [117, 118]. Furthermore, there is a growing body of evidence that suggests that the heterogeneity of MSCs may contribute to their broad therapeutic efficacy, with multiple cell populations participating in tissue repair through diverse mechanisms that include the regulation of inflammation and apoptosis. Frequently these repair mechanisms are examined with the entire MSC preparation rather than its constituent populations [80].

It is not really known whether these subpopulations are endowed with different engraftment capacity in the lung. The heterogeneity of marrow cells and their plasticity in relation to the lung has been recently appreciated in the mouse using adherent subpopulations expressing both hematopoietic markers (CD34, CD45) and mesenchymal markers (CD73, CD90, CD105) and by differentiating them on the basis of the expression of Clara cell secretory protein (CcsP) mRNA and protein [119]. More Ccsp^+^ cells were found in naphthalene-injured lungs compared with Ccsp^−^ cells when delivered transtracheally to the airways. Ccsp^+^ cells were able to contribute to the reconstitution of injured airways by 56% and 25% at 32 and 62 days after bone marrow transplantation, respectively. Which stage in the development of marrow cells do these cells represent is difficult to say, also because they have only some features in common with other subpopulations identified in the murine bone marrow [120, 121]. It is noteworthy to mention that several studies in mice have shown that nonadherent HSPCs that can reconstitute the blood are also capable to give rise to osteoblasts [122, 123]. The Krause group has demonstrated in mice that nonhematopoietic (lin^−^) BM cells are the primary source of donor-derived lung epithelial cells by showing that they consistently give rise to surfactant protein C (SPC) positive lung epithelial cells in SPC-knockout recipient mice, while hematopoietic BM cells do not [124]. Further studies in mice, using imaging flow cytometry, which allows for single cell analysis without interference of cell overlay or background fluorescence, have determined that the subpopulation of nonhematopoietic cells in adult BM that is able to give rise to lung epithelial cells is composed of small embryonic-like cells (VSELs) [125]. These cells are small (2–6 *μ*m), lin^−^, CD45^−^, positive for Oct4 and Nanog, and give rise to cells of three germ layer lineages *in vitro* [126]. Other marrow-derived stem cell subpopulations have been described in mice and their main features are given in Table 1.

Our recently published data confirm the HSPC heterogeneity in both lin^−^ Sca-1^+^ and positively selected Sca-1^+^ murine HSPCs and suggest the role that mitochondria can play in HSPC biology [128]. In an attempt to differentiate hematopoietic progenitor cells on the basis of their phenotype and mitochondrial content profile (as studied by staining with MitoTracker Green [MTG], a dye which enters into functional mitochondria), we identified two populations: a population of smaller cells with a higher percentage of Sca-1^+^ and lower MTG^+^ signal (R1) and a population of bigger cells containing a lower percentage of Sca-1^+^ and higher MTG^+^ (R2). These two populations also showed a different CFTR expression which was higher in the subset with the bigger size, although the CFTR expression was quite low in both subsets (11% and 15% in smaller and bigger cells, resp.). Since murine HSPCs lose Sca-1 upon commitment to myeloid and lymphoid progenitors [129, 130], our data indicate that mitochondrial biogenesis is linked to and likely required in the first steps of stem/progenitor cell differentiation, as it was suggested for human CD34^+^ cells [131]. Furthermore, the low CFTR expression detected by us in both subpopulations might explain why wild type marrow cells transplanted in CF mice did not have a strong therapeutic effect. However, we could establish that R1 is the murine subpopulation which is endowed with the highest chemotactic activity in the presence of an SDF-1 gradient [132]. The relationship between R1 (smaller) and R2 (bigger) subpopulations with Ccsp^+^ and Ccsp^−^ cells [119] is not known at the moment. Ccsp^+^ cells are small (~5–10 *μ*m) and round cells and express Sca-1. In contrast, Ccsp^−^ cells (>10 *μ*m) range from very large cells with a lot of cytoplasm to medium-sized cells. It is tempting to speculate that R1 cells are similar either to Ccsp^+^ cells or to VSELs, which show the highest regenerative potential in the lung, and for this reason, expression of Ccsp and hematopoietic/mesenchymal markers will be studied in the near future, as well as the differentiative properties of R1 cells towards hematopoietic, mesenchymal, and epithelial lineages. Overall, these studies highlight the presence of rare populations of epithelial stem/progenitor cells in the bone marrow. Their potential is still to be exploited in animal models, above all for the effects on lung structure and mechanics [29].

Instead of a hierarchical model, some studies suggest that regulation of HPSCs is on a continuum [133]. In other words, the phenotype of murine HSPCs reversibly varies with the stage of cell cycle, and this conversion is likely due to chromatin access for transcription factors which varies in response to differentiating inducing stimuli [134]. This has led to investigate whether the murine HSPCs labile state in regarding to cell cycle has an implication for their capacity of homing to the lung and give rise to differentiated epithelial cells [135]. Lin^−^ Sca^+^ HSPCs cultured with IL-3, IL-6 and IL-11 for 24 hours (and entered in the G1/S interface), showed three-fold increase in pulmonary epithelial cells as compared with freshly isolated cells upon transplantation in injured mice. This increased capacity to make lung cells had returned to baseline at 48 hours of culture. Regarding the efficiency of transformation of marrow cells into respiratory epithelial cells, these results could convey the notion that stimulating the proliferation of marrow cells would lead to an enhancement of the epithelial cell regenerative capacity. On the other hand, concerning the mechanism(s) behind the phenomenon, it could be that cytokines are mimicking signals coming from the damaged lung. It has been proposed that chemokines, such as SDF-1 (or CXCL12), are the main stimuli which attract circulating CXCR4-expressing epithelial progenitor cells to the lung in rodents and humans [56, 136, 137].

## 6. Conclusion and Perspectives

The application of marrow-derived stem and progenitor cells to chronic lung diseases, and as described in this review, is still in its infancy. Many parameters should be considered, which have not been fully analyzed in the many studies on this subject, including different subsets of cells and the signal(s) involved in their homing to the injured and repairing lung. It might well be that the functional heterogeneity of subpopulations is reflected also by the different capacities of progenitor/stem cells to home to epithelia-lined organs and transform into epithelial cells, as it has been previously demonstrated [119].

A caveat to these studies is safety. It could be that the inflammatory response in the lung tissue is aggravated by differentiation of BM-derived stem/progenitor cells into mature myeloid cells. This is suggested by recent work in mice [138], which demonstrated that bone marrow transplantation contributes to lung inflammation in CF mice by giving rise to lung macrophages. Inflammatory cells (neutrophils, macrophages) also contribute to the remodelling of lung by secreting proteases, including elastase and metalloproteases. Finally, a control on the transplanted cells could be important for safety reasons. As it has been done with allogeneic bone marrow transplantation in leukemic patients with the suicide gene therapy, the transplanted cells may be engineered with the Herpes simplex-virus thymidine kinase (TK) gene and controlled by the administration of a nontoxic prodrug-like ganciclovir [139]. It should be emphasized that two major limitations have emerged in clinical trials with TK cells, namely, expression of nonfunctional TK molecules and the immunogenicity of viral-derived TK protein, which have limited the usefulness of this approach with BM transplantation studies in humans [140]. Alternatively, marrow cells might be administered via local injection [51, 52], thereby bypassing the intravascular milieu which could facilitate or induce their differentiation into neutrophils or macrophages. As a third option, HSPCs could be coadministered along with an MSC population. As discussed above, intrapulmonary administration of MSCs attenuated inflammation and lung injury despite minimal, if any, engraftment of MSCs in the lung of mouse and rat models [78, 79, 141], an effect explained by paracrine effects due to the release of anti-inflammatory mediators.

In short, continuous effort has to be dedicated to overcome technical limitations in identifying marrow-derived lung epithelial cells, to elucidate possible mechanism(s) underlying the positive role of marrow cells in repairing lung injury and to identify subpopulations which could engraft in the lung. This knowledge could ameliorate marrow-derived stem cell homing to the lung and their acquisition of lung cell phenotype, as well as the modulation of inflammatory and immune responses, obtaining a therapeutic outcome in such deadly respiratory diseases.

## Figures and Tables

**Table 1:** Characteristics of marrow-derived stem cell subpopulations in murine models.

Subpopulation	Size	Phenotype	Marrow-derived epithelial cells in the lung	Reference
ELH^1^	<5 *μ*m	Lin^−^	Yes	[26]
LSK^2^	ND	Lin^−^, Sca-1^+^, Kit^+^	No	[45]
MAPCs^3^	8–10 *μ*m	Flk-1^+^, Sca-1^+^, Thy-1^+^, CD13^+^, SSEA-1^+^	Yes	[53]
SSEA-1^4^		Lin^−^, CD45^−^, CD31^+^, Sca-1^+^, CD105^+^, CD73^+^, CD44^+^, vimentin^+^	ND	[127]
VSELs^5^	2–4 *μ*m	Lin^−^, Sca-1^+^, CD45^−^, SSEA-1^+^, Oct-4^+^, Nanog^+^	Yes	[125]
Ccsp^6^	5–10 *μ*m	CD45^+^, CD34^+^, CD73^+^, CD90^+^, CD105^+^	Yes	[119]
R1/R2 CFTR^7^	ND	Lin^−^, Sca-1^+^, CFTR^+^	ND	[128]

^1^Elutriation (E), lineage depletion (L), ability to home (H) to the BM; ^2^Lineage-negative (L), Sca^+^ (S), Kit^+^ (K); ^3^Multipotent adult progenitor cells; ^4^Stage-specific embryonic antigen 1; ^5^Very small, embryonic-like cells; ^6^Clara cell secretory protein; ^7^Cystic fibrosis transmembrane conductance regulator. ND: not determined.

## References

[1] Murray CJL, Lopez AD (1997). Alternative projections of mortality and disability by cause 1990–2020: global Burden of Disease Study. *The Lancet*.

[2] Barnes PJ (2013). Corticosteroid resistance in patients with asthma and chronic obstructive pulmonary disease. *Journal of Allergy and Clinical Immunology*.

[3] Becq F (2010). Cystic fibrosis transmembrane conductance regulator modulators for personalized drug treatment of cystic fibrosis: progress to date. *Drugs*.

[4] Grove JE, Bruscia E, Krause DS (2004). Plasticity of bone marrow-derived stem cells. *Stem Cells*.

[5] Denburg JA, van Eeden SF (2006). Bone marrow progenitors in inflammation and repair: new vistas in respiratory biology and pathophysiology. *European Respiratory Journal*.

[6] Weiss DJ, Berberich MA, Borok Z (2006). Adult stem cells, lung biology, and lung disease. *Proceedings of the American Thoracic Society*.

[7] Gomperts BN, Strieter RM (2007). Stem cells and chronic lung disease. *Annual Review of Medicine*.

[8] Weiss DJ, Kolls JK, Ortiz LA, Panoskaltsis-Mortari A, Prockop DJ (2008). Stem cells and cell therapies in lung biology and lung diseases. *Proceedings of the American Thoracic Society*.

[9] Sueblinvong V, Weiss DJ (2010). Stem cells and cell therapy approaches in lung biology and diseases. *Translational Research*.

[10] Pesci A, Balbi B, Majori M (1998). Inflammatory cells and mediators in bronchial lavage of patients with chronic obstructive pulmonary disease. *European Respiratory Journal*.

[11] Barnes PJ (2000). Chronic obstructive pulmonary disease. *The New England Journal of Medicine*.

[12] Fischer BM, Pavlisko E, Voynow JA (2011). Pathogenic triad in COPD: oxidative stress, protease-antiprotease imbalance, and inflammation. *International Journal of Chronic Obstructive Pulmonary Disease*.

[13] Sahiner UM, Birben E, Erzurum S, Sackesen C, Kalayci O (2011). Oxidative stress in asthma. *World Allergy Organization Journal*.

[14] Barnes PJ (2011). Pathophysiology of allergic inflammation. *Immunological Reviews*.

[15] Aoshiba K, Nagai A (2004). Differences in airway remodeling between asthma and chronic obstructive pulmonary disease. *Clinical Reviews in Allergy and Immunology*.

[16] Akabas MH (2000). Cystic fibrosis transmembrane conductance regulator. Structure and function of an epithelial chloride channel. *Journal of Biological Chemistry*.

[17] Boucher RC (2007). Cystic fibrosis: a disease of vulnerability to airway surface dehydration. *Trends in Molecular Medicine*.

[18] Rowe SM, Miller S, Sorscher EJ (2005). Cystic fibrosis. *The New England Journal of Medicine*.

[19] Hartl D, Latzin P, Hordijk P (2007). Cleavage of CXCR1 on neutrophils disables bacterial killing in cystic fibrosis lung disease. *Nature Medicine*.

[20] Conese M (2011). Cystic fibrosis and the innate immune system: therapeutic implications. *Endocrine, Metabolic and Immune Disorders*.

[21] Di A, Brown ME, Deriy LV (2006). CFTR regulates phagosome acidification in macrophages and alters bactericidal activity. *Nature Cell Biology*.

[22] Zhang Y, Li X, Grassmé H, Döring G, Gulbins E (2010). Alterations in ceramide concentration and pH determine the release of reactive oxygen species by Cftr-deficient macrophages on infection. *Journal of Immunology*.

[23] Hilliard TN, Regamey N, Shute JK (2007). Airway remodelling in children with cystic fibrosis. *Thorax*.

[24] Ulrich M, Worlitzsch D, Viglio S (2010). Alveolar inflammation in cystic fibrosis. *Journal of Cystic Fibrosis*.

[25] Gaggar A, Hector A, Bratcher PE, Mall MA, Griese M, Hartl D (2011). Series “matrix metalloproteinases in lung health and disease”: the role of matrix metalloproteinases in cystic fibrosis lung disease. *European Respiratory Journal*.

[26] Krause DS, Theise ND, Collector MI (2001). Multi-organ, multi-lineage engraftment by a single bone marrow-derived stem cell. *Cell*.

[27] Wong AP, Dutly AE, Sacher A (2007). Targeted cell replacement with bone marrow cells for airway epithelial regeneration. *American Journal of Physiology—Lung Cellular and Molecular Physiology*.

[28] Tropea KA, Leder E, Aslam M (2012). Bronchioalveolar stem cells increase after mesenchymal stromal cell treatment in a mouse model of bronchopulmonary dysplasia. *American Journal of Physiology—Lung Cellular and Molecular Physiology*.

[29] Eisenhauer P, Earle B, Loi R (2013). Endogenous distal airway progenitor cells, lung mechanics, and disproportionate lobar growth following long-term postpneumonectomy in mice. *Stem Cells*.

[30] Abe S, Boyer C, Liu X (2004). Cells derived from the circulation contribute to the repair of lung injury. *American Journal of Respiratory and Critical Care Medicine*.

[31] Theise ND, Henegariu O, Grove J (2002). Radiation pneumonitis in mice: a severe injury model for pneumocyte engraftment from bone marrow. *Experimental Hematology*.

[32] Grove JE, Lutzko C, Priller J (2002). Marrow-derived cells as vehicles for delivery of gene therapy to pulmonary epithelium. *American Journal of Respiratory Cell and Molecular Biology*.

[33] Serikov VB, Popov B, Mikhailov VM, Gupta N, Matthay MA (2007). Evidence of temporary airway epithelial repopulation and rare clonal formation by BM-derived cells following naphthalene injury in mice. *Anatomical Record*.

[34] Suratt BT, Cool CD, Serls AE (2003). Human pulmonary chimerism after hematopoietic stem cell transplantation. *American Journal of Respiratory and Critical Care Medicine*.

[35] Mattsson J, Jansson M, Wernerson A, Hassan M (2004). Lung epithelial cells and type II pneumocytes of donor origin after allogeneic hematopoietic stem cell tansplantation. *Transplantation*.

[36] Kleeberger W, Versmold A, Rothämel T (2003). Increased chimerism of bronchial and alveolar epithelium in human lung allografts undergoing chronic injury. *American Journal of Pathology*.

[37] Weiss DJ, Bertoncello I, Borok Z (2011). Stem cells and cell therapies in lung biology and lung diseases. *Proceedings of the American Thoracic Society*.

[38] Aliotta JM, Keaney P, Passero M (2006). Bone marrow production of lung cells: the impact of G-CSF, cardiotoxin, graded doses of irradiation, and subpopulation phenotype. *Experimental Hematology*.

[39] Herzog EL, Van Arnam J, Hu B, Krause DS (2006). Threshold of lung injury required for the appearance of marrow-derived lung epithelia. *Stem Cells*.

[40] Conese M, Copreni E, Piro D, Rejman J (2007). Gene and cell therapy for the treatment of cystic fibrosis. *Advances in Gene, Molecular and Cell Therapy*.

[41] MacPherson H, Keir PA, Edwards CJ, Webb S, Dorin JR (2006). Following damage, the majority of bone marrow-derived airway cells express an epithelial marker. *Respiratory Research*.

[42] Rojas M, Xu J, Woods CR (2005). Bone marrow-derived mesenchymal stem cells in repair of the injured lung. *American Journal of Respiratory Cell and Molecular Biology*.

[43] Kotton DN, Ma BY, Cardoso WV (2001). Bone marrow-derived cells as progenitors of lung alveolar epithelium. *Development*.

[44] Kotton DN, Fabian AJ, Mulligan RC (2005). Failure of bone marrow to reconstitute lung epithelium. *American Journal of Respiratory Cell and Molecular Biology*.

[45] Wagers AJ, Sherwood RI, Christensen JL, Weissman IL (2002). Little evidence for developmental plasticity of adult hematopoietic stem cells. *Science*.

[46] Chang JC, Summer R, Sun X, Fitzsimmons K, Fine A (2005). Evidence that bone marrow cells do not contribute to the alveolar epithelium. *American Journal of Respiratory Cell and Molecular Biology*.

[47] Fritzell JA, Mao Q, Gundavarapu S (2009). Fate and effects of adult bone marrow cells in lungs of normoxic and hyperoxic newborn mice. *American Journal of Respiratory Cell and Molecular Biology*.

[48] Quesenberry PJ, Colvin G, Dooner G, Dooner M, Aliotta JM, Johnson K (2007). The stem cell continuum: cell cycle, injury, and phenotype lability. *Annals of the New York Academy of Sciences*.

[49] Krause DS (2008). Bone marrow-derived lung epithelial cells. *Proceedings of the American Thoracic Society*.

[50] Kassmer SH, Krause DS (2010). Detection of bone marrow-derived lung epithelial cells. *Experimental Hematology*.

[51] Leblond A-L, Naud P, Forest V (2009). Developing cell therapy techniques for respiratory disease: intratracheal delivery of genetically engineered stem cells in a murine model of airway injury. *Human Gene Therapy*.

[52] Rejman J, Colombo C, Conese M (2009). Engraftment of bone marrow-derived stem cells to the lung in a model of acute respiratory infection by Pseudomonas aeruginosa. *Molecular Therapy*.

[53] Jiang Y, Jahagirdar BN, Reinhardt RL (2002). Pluripotency of mesenchymal stem cells derived from adult marrow. *Nature*.

[54] Kucia M, Reca R, Jala VR, Dawn B, Ratajczak J, Ratajczak MZ (2005). Bone marrow as a home of heterogenous populations of nonhematopoietic stem cells. *Leukemia*.

[55] Kucia M, Ratajczak J, Ratajczak MZ (2005). Bone marrow as a source of circulating CXCR4+ tissue-committed stem cells. *Biology of the Cell*.

[56] Gomperts BN, Belperio JA, Rao PN (2006). Circulating progenitor epithelial cells traffic via CXCR4/CXCL12 in response to airway injury. *Journal of Immunology*.

[57] Harris RG, Herzog EL, Bruscia EM, Grove JE, Van Arnam JS, Krause DS (2004). Lack of a fusion requirement for development of bone marrow-derived epithelia. *Science*.

[58] Alvarez-Dolado M, Pardal R, Garcia-Verdugo JM (2003). Fusion of bone-marrow-derived cells with Purkinje neurons, cardiomyocytes and hepatocytes. *Nature*.

[59] Herzog EL, Van Arnam J, Hu B (2007). Lung-specific nuclear reprogramming is accompanied by heterokaryon formation and Y chromosome loss following bone marrow transplantation and secondary inflammation. *FASEB Journal*.

[60] Aliotta JM, Sanchez-Guijo FM, Dooner GJ (2007). Alteration of marrow cell gene expression, protein production, and engraftment into lung by lung-derived microvesicles: a novel mechanism for phenotype modulation. *Stem Cells*.

[61] Aliotta JM, Pereira M, Johnson KW (2010). Microvesicle entry into marrow cells mediates tissue-specific changes in mRNA by direct delivery of mRNA and induction of transcription. *Experimental Hematology*.

[62] Lau AN, Goodwin M, Kim CF, Weiss DJ (2012). Stem cells and regenerative medicine in lung biology and diseases. *Molecular Therapy*.

[63] Conese M, Carbone A, Castellani S, Di Gioia S (2013). Paracrine effects and heterogeneity of marrow-derived stem/progenitor cells: relevance for the treatment of respiratory diseases. *Cells Tissues Organs*.

[64] Yamada M, Kubo H, Kobayashi S (2004). Bone marrow-derived progenitor cells are important for lung repair after lipopolysaccharide-induced lung injury. *Journal of Immunology*.

[65] Wang X-X, Zhang F-R, Shang Y-P (2007). Transplantation of autologous endothelial progenitor cells may be beneficial in patients with idiopathic pulmonary arterial hypertension. A pilot randomized controlled trial. *Journal of the American College of Cardiology*.

[66] Zhu JH, Wang XX, Zhang FR (2008). Safety and efficacy of autologous endothelial progenitor cells transplantation in children with idiopathic pulmonary arterial hypertension: open-label pilot study. *Pediatric Transplantation*.

[67] Diller G-P, Thum T, Wilkins MR, Wharton J (2010). Endothelial progenitor cells in pulmonary arterial hypertension. *Trends in Cardiovascular Medicine*.

[68] Phillips RJ, Burdick MD, Hong K (2004). Circulating fibrocytes traffic to the lungs in response to CXCL12 and mediate fibrosis. *Journal of Clinical Investigation*.

[69] Epperly MW, Guo H, Gretton JE, Greenberger JS (2003). Bone marrow origin of myofibroblasts in irradiation pulmonary fibrosis. *American Journal of Respiratory Cell and Molecular Biology*.

[70] Hashimoto N, Jin H, Liu T, Chensue SW, Phan SH (2004). Bone marrow-derived progenitor cells in pulmonary fibrosis. *Journal of Clinical Investigation*.

[71] Moore BB, Kolodsick JE, Thannickal VJ (2005). CCR2-mediated recruitment of fibrocytes to the alveolar space after fibrotic injury. *American Journal of Pathology*.

[72] Tanjore H, Xu XC, Polosukhin VV (2009). Contribution of epithelial-derived fibroblasts to bleomycin-induced lung fibrosis. *American Journal of Respiratory and Critical Care Medicine*.

[73] Strieter RM, Keeley EC, Burdick MD, Mehrad B (2009). The role of circulating mesenchymal progenitor cells, fibrocytes, in promoting pulmonary fibrosis. *Transactions of the American Clinical and Climatological Association*.

[74] Keeley EC, Mehrad B, Strieter RM (2010). Fibrocytes: bringing new insights into mechanisms of inflammation and fibrosis. *International Journal of Biochemistry and Cell Biology*.

[75] Gomperts BN, Strieter RM (2007). Fibrocytes in lung disease. *Journal of Leukocyte Biology*.

[76] Bellini A, Mattoli S (2007). The role of the fibrocyte, a bone marrow-derived mesenchymal progenitor, in reactive and reparative fibroses. *Laboratory Investigation*.

[77] Ortiz LA, Gambelli F, McBride C (2003). Mesenchymal stem cell engraftment in lung is enhanced in response to bleomycin exposure and ameliorates its fibrotic effects. *Proceedings of the National Academy of Sciences of the United States of America*.

[78] Gupta N, Su X, Popov B, Jae WL, Serikov V, Matthay MA (2007). Intrapulmonary delivery of bone marrow-derived mesenchymal stem cells improves survival and attenuates endotoxin-induced acute lung injury in mice. *Journal of Immunology*.

[79] Xu J, Woods CR, Mora AL (2007). Prevention of endotoxin-induced systemic response by bone marrow-derived mesenchymal stem cells in mice. *American Journal of Physiology—Lung Cellular and Molecular Physiology*.

[80] Ortiz LA, DuTreil M, Fattman C (2007). Interleukin 1 receptor antagonist mediates the antiinflammatory and antifibrotic effect of mesenchymal stem cells during lung injury. *Proceedings of the National Academy of Sciences of the United States of America*.

[81] Chamberlain G, Fox J, Ashton B, Middleton J (2007). Concise review: mesenchymal stem cells: their phenotype, differentiation capacity, immunological features, and potential for homing. *Stem Cells*.

[82] Rasmusson I (2006). Immune modulation by mesenchymal stem cells. *Experimental Cell Research*.

[83] Duffy MM, Ritter T, Ceredig R, Griffin MD (2011). Mesenchymal stem cell effects on T-cell effector pathways. *Stem Cell Research and Therapy*.

[84] Bifari F, Lisi V, Mimiola E, Pasini A, Krampera M (2008). Immune modulation by mesenchymal stem cells. *Transfusion Medicine and Hemotherapy*.

[85] Le Blanc K, Frassoni F, Ball L (2008). Mesenchymal stem cells for treatment of steroid-resistant, severe, acute graft-versus-host disease: a phase II study. *The Lancet*.

[86] Duijvestein M, Vos ACW, Roelofs H (2010). Autologous bone marrow-derived mesenchymal stromal cell treatment for refractory luminal Crohn’s disease: results of a phase I study. *Gut*.

[87] Salem HK, Thiemermann C (2010). Mesenchymal stromal cells: current understanding and clinical status. *Stem Cells*.

[88] Abreu SC, Antunes MA, Pelosi P, Morales MM, Rocco PRM (2011). Mechanisms of cellular therapy in respiratory diseases. *Intensive Care Medicine*.

[89] de Faria CA, de las Heras Kozma R, Stessuk T, Ribeiro-Paes JT (2012). Experimental basis and new insights for cell therapy in chronic obstructive pulmonary disease. *Stem Cell Reviews*.

[90] Loi R, Beckett T, Goncz KK, Suratt BT, Weiss DJ (2006). Limited restoration of cystic fibrosis lung epithelium in vivo with adult bone marrow-derived cells. *American Journal of Respiratory and Critical Care Medicine*.

[91] Bruscia EM, Ziegler EC, Price JE, Weiner S, Egan ME, Krause DS (2006). Engraftment of donor-derived epithelial cells in multiple organs following bone marrow transplantation into newborn mice. *Stem Cells*.

[92] Bruscia EM, Price JE, Cheng E-C (2006). Assessment of cystic fibrosis transmembrane conductance regulator (CFTR) activity in CFTR-null mice after bone marrow transplantation. *Proceedings of the National Academy of Sciences of the United States of America*.

[93] Agostini C (2010). Stem cell therapy for chronic lung diseases: hope and reality. *Respiratory Medicine*.

[94] Ribeiro-Paes JT, Bilaqui A, Greco OT (2011). Unicentric study of cell therapy in chronic obstructive pulmonary disease/pulmonary emphysema. *International Journal of COPD*.

[95] Weiss DJ, Casaburi R, Flannery R, Leroux-Williams M, Tashkin DP (2013). A placebo-controlled randomized trial of mesenchymal stem cells in chronic obstructive pulmonary disease. *Chest*.

[96] Spangrude GJ, Johnson GR (1990). Resting and activated subsets of mouse multipotent hematopoietic stem cells. *Proceedings of the National Academy of Sciences of the United States of America*.

[97] Jones RJ, Collector MI, Barber JP (1996). Characterization of mouse lymphohematopoietic stem cells lacking spleen colony-forming activity. *Blood*.

[98] Jordan CT, Yamasaki G, Minamoto D (1996). High-resolution cell cycle analysis of defined phenotypic subsets within primitive human hematopoietic cell populations. *Experimental Hematology*.

[99] Uchida N, Jerabek L, Weissman IL (1996). Searching for hematopoietic stem cells. II. The heterogeneity of Thy-1.1(lo)Lin(-/lo)Sca-1+ mouse hematopoietic stem cells separated by counterflow centrifugal elutriation. *Experimental Hematology*.

[100] Goodell MA, Rosenzweig M, Kim H (1997). Dye efflux studies suggest that hematopoietic stem cells expressing low or undetectable levels of CD34 antigen exist in multiple species. *Nature Medicine*.

[101] Gothot A, Pyatt R, McMahel J, Rice S, Srour EF (1997). Functional heterogeneity of human CD34^+^ cells isolated in subcompartments of the G0/G1 phase of the cell cycle. *Blood*.

[102] Gothot A, Pyatt R, McMahel J, Rice S, Srour EF (1998). Assessment of proliferative and colony-forming capacity after successive in vitro divisions of single human CD34^+^ cells initially isolated in G0. *Experimental Hematology*.

[103] Wiesmann A, Phillips RL, Mojica M (2000). Expression of CD27 on murine hematopoietic stem and progenitor cells. *Immunity*.

[104] Dykstra B, Kent D, Bowie M (2007). Long-term propagation of distinct hematopoietic differentiation programs in vivo. *Cell Stem Cell*.

[105] Kent DG, Copley MR, Benz C (2009). Prospective isolation and molecular characterization of hematopoietic stem cells with durable self-renewal potential. *Blood*.

[106] Sieburg HB, Cho RH, Dykstra B, Uchida N, Eaves CJ, Muller-Sieburg CE (2006). The hematopoietic stem compartment consists of a limited number of discrete stem cell subsets. *Blood*.

[107] Benveniste P, Frelin C, Janmohamed S (2010). Intermediate-term hematopoietic stem cells with extended but time-limited reconstitution potential. *Cell Stem Cell*.

[108] Lemischka IR, Raulet RH, Mulligan RC (1986). Developmental potential and dynamic behavior of hematopoietic stem cells. *Cell*.

[109] Challen GA, Boles NC, Chambers SM, Goodell MA (2010). Distinct hematopoietic stem cell subtypes are differentially regulated by TGF-*β*1. *Cell Stem Cell*.

[110] Schroeder T (2010). Hematopoietic stem cell heterogeneity: subtypes, not unpredictable behavior. *Cell Stem Cell*.

[111] Cross MA, Heyworth CM, Murrell AM, Bockamp EO, Dexter TM, Green AR (1994). Expression of lineage restricted transcription factors precedes lineage specific differentiation in a multipotent haemopoietic progenitor cell line. *Oncogene*.

[112] Chang HH, Hemberg M, Barahona M, Ingber DE, Huang S (2008). Transcriptome-wide noise controls lineage choice in mammalian progenitor cells. *Nature*.

[113] Graf T, Enver T (2009). Forcing cells to change lineages. *Nature*.

[114] Schroeder T (2011). Long-term single-cell imaging of mammalian stem cells. *Nature Methods*.

[115] Hope K, Bhatia M (2011). Clonal interrogation of stem cells. *Nature Methods*.

[116] Phinney DG (2007). Biochemical heterogeneity of mesenchymal stem cell populations: clues to their therapeutic efficacy. *Cell Cycle*.

[117] Russell KC, Phinney DG, Lacey MR, Barrilleaux BL, Meyertholen KE, O’Connor KC (2010). In vitro high-capacity assay to quantify the clonal heterogeneity in trilineage potential of mesenchymal stem cells reveals a complex hierarchy of lineage commitment. *Stem Cells*.

[118] Russell KC, Lacey MR, Gilliam JK, Tucker HA, Phinney DG, O’Connor KC (2011). Clonal analysis of the proliferation potential of human bone marrow mesenchymal stem cells as a function of potency. *Biotechnology and Bioengineering*.

[119] Wong AP, Keating A, Lu W-Y (2009). Identification of a bone marrow-derived epithelial-like population capable of repopulating injured mouse airway epithelium. *Journal of Clinical Investigation*.

[120] Kucia M, Wysoczynski M, Ratajczak J, Ratajczak MZ (2008). Identification of very small embryonic like (VSEL) stem cells in bone marrow. *Cell and Tissue Research*.

[121] Ratajczak J, Wysoczynski M, Zuba-Surma E (2011). Adult murine bone marrow-derived very small embryonic-like stem cells differentiate into the hematopoietic lineage after coculture over OP9 stromal cells. *Experimental Hematology*.

[122] Dominici M, Pritchard C, Garlits JE, Hofmann TJ, Persons DA, Horwitz EM (2004). Hematopoietic cells and osteoblasts are derived from a common marrow progenitor after bone marrow transplantation. *Proceedings of the National Academy of Sciences of the United States of America*.

[123] Olmsted-Davis EA, Gugala Z, Camargo F (2003). Primitive adult hematopoietic stem cells can function as osteoblast precursors. *Proceedings of the National Academy of Sciences of the United States of America*.

[124] Kassmer SH, Bruscia EM, Zhang P-X, Krause DS (2012). Nonhematopoietic cells are the primary source of bone marrow-derived lung epithelial cells. *Stem Cells*.

[125] Kassmer SH, Jin H, Zhang PX (2013). Very small embryonic-like stem cells from the murine bone marrow differentiate into epithelial cells of the lung. *Stem Cells*.

[126] Kucia M, Reca R, Campbell FR (2006). A population of very small embryonic-like (VSEL) CXCR4+ SSEA-1+Oct-4+ stem cells identified in adult bone marrow. *Leukemia*.

[127] Anjos-Afonso F, Bonnet D (2007). Nonhematopoietic/endothelial SSEA-1+ cells define the most primitive progenitors in the adult murine bone marrow mesenchymal compartment. *Blood*.

[128] Piro D, Piccoli C, Guerra L (2012). Hematopoietic stem/progenitor cells express functional mitochondrial energy-dependent cystic fibrosis transmembrane conductance regulator. *Stem Cells and Development*.

[129] Akashi K, Traver D, Miyamoto T, Weissman IL (2000). A clonogenic common myeloid progenitor that gives rise to all myeloid lineages. *Nature*.

[130] Kondo M, Weissman IL, Akashi K (1997). Identification of clonogenic common lymphoid progenitors in mouse bone marrow. *Cell*.

[131] Piccoli C, Ria R, Scrima R (2005). Characterization of mitochondrial and extra-mitochondrial oxygen consuming reactions in human hematopoietic stem cells: novel evidence of the occurrence of NAD(P)H oxidase activity. *Journal of Biological Chemistry*.

[132] Trotta T, Di Gioia S, Piro D (2013). Effect of acute lung injury on VLA-4 and CXCR4 expression in resident and circulating hematopoietic stem/progenitor cells. *Respiration*.

[133] Quesenberry PJ (2006). The continuum model of marrow stem cell regulation. *Current Opinion in Hematology*.

[134] Colvin GA, Dooner MS, Dooner GJ (2007). Stem cell continuum: directed differentiation hotspots. *Experimental Hematology*.

[135] Dooner MS, Aliotta JM, Pimentel J (2008). Conversion potential of marrow cells into lung cells fluctuates with cytokine-induced cell cycle. *Stem Cells and Development*.

[136] Xu J, Mora A, Shim H, Stecenko A, Brigham KL, Rojas M (2007). Role of the SDF-1/CXCR4 axis in the pathogenesis of lung injury and fibrosis. *American Journal of Respiratory Cell and Molecular Biology*.

[137] May LA, Kicic A, Rigby P (2009). Cells of epithelial lineage are present in blood, engraft the bronchial epithelium, and are increased in human lung transplantation. *Journal of Heart and Lung Transplantation*.

[138] Bruscia EM, Zhang P-X, Ferreira E (2009). Macrophages directly contribute to the exaggerated inflammatory response in cystic fibrosis transmembrane conductance regulator-/-mice. *American Journal of Respiratory Cell and Molecular Biology*.

[139] Bonini C, Ferrari G, Verzeletti S (1997). HSV-TK gene transfer into donor lymphocytes for control of allogeneic graft-versus-leukemia. *Science*.

[140] Mastaglio S, Stanghellini MTL, Bordignon C, Bondanza A, Ciceri F, Bonini C (2010). Progress and prospects: graft-versus-host disease. *Gene Therapy*.

[141] Zhao F, Zhang YF, Liu YG (2008). Therapeutic effects of bone marrow-derived mesenchymal stem cells engraftment on bleomycin-induced lung injury in rats. *Transplantation Proceedings*.

